# Spiritual Health and Its Determinants Among Urban Adolescents in Northern India: A Cross-Sectional Survey

**DOI:** 10.7759/cureus.58609

**Published:** 2024-04-19

**Authors:** Souvik Manna, Arun Udayaraj, Sumit Grover, Vinod Kumar

**Affiliations:** 1 Community Medicine, Employees State Insurance Corporation (ESIC) Medical College and Hospital, Alwar, Alwar, IND; 2 Internal Medicine, Employees State Insurance Corporation (ESIC) Medical College and Hospital, Alwar, Alwar, IND; 3 Ophthalmology, National Cancer Institute, All India Institute of Medical Sciences, New Delhi, New Delhi, IND; 4 Community Ophthalmology, All India Institute of Medical Sciences, New Delhi, New Delhi, IND

**Keywords:** positive health, spirituality, youth, spiritual health, positive youth development, adolescent health

## Abstract

Introduction: Spiritual health is an important dimension of positive health and is often ignored as it is not amenable to measurement. The present study was conducted to generate relevant evidence on spiritual health among adolescents living in urban areas of Northern India.

Methods: A cross-sectional study was done from June 2019 - May 2020 in an urban area of Northern India on a sample of 300 adolescents selected purposively. After collection of demographic details of the participants, the Index of Core Spiritual Experiences (INSPIRIT) tool was used to capture their spiritual health.

Results: The Cronbach’s alpha for the scale was 0.832 (0.797-0.863) indicating good internal consistency of the measure. As far as spiritual health is concerned, 217 (72.3%) of the study participants scored medium-high to high, followed by 83 (27.7%) who scored medium-low to low on the spiritual health scale. Adjusted multivariate analysis using binary logistic regression showed that positive traits like caring (odds ratio (OR) 1.19, 95% CI: 1.08-1.33), connection to school (OR 1.16, 95% CI: 1.04-1.29), having positive identity (OR 1.19, 95% CI: 1.04-1.36) and having highly educated (post-graduate) parents (OR 2.18, 95% CI: 1.13-4.21) lead to significantly higher spiritual health scores.

Discussion: Although spiritual health is not routinely measured among adolescents, the current study demonstrated high levels of spiritual health among half of the urban adolescents. Parental education was found to have a positive association with spiritual health scores, indicating the indirect effect of parental spiritual inclination. The study has important implications for policy, as it demonstrates the feasibility of measuring a covert dimension of health which tends to have an indirect effect on holistic youth development.

## Introduction

“Health is a state of complete physical, mental and social well-being and not merely an absence of disease or infirmity, and also the ability to lead a socially and economically productive life’’ [1]. This World Health Organization (WHO) definition envisages three specific dimensions- the physical, the mental and the social. Many more may be cited, like spiritual, emotional, vocational and political dimensions [2]. The WHO has also identified spirituality/religion/personal beliefs as one of the six broad domains of quality of life. Thus, spiritual health is an important determinant of quality of life. According to the existing literature, spiritual health is defined as connection with God (a superior existence), oneself, others and nature. The current study focussed on the spiritual health of adolescents, which is much lacking in research. Spiritual health is an important determinant of both quality of health as well as mental health and can be considered as an important intervention for youth development.

WHO defines adolescents as individuals between the ages of 10 to 19 years. Youth is defined by the United Nations as persons between the ages of 15 and 24; it is a transitional period from childhood to adulthood. The young are often among the most vulnerable or disadvantaged in society and their quality of life requires special attention. Worldwide, children younger than age 18 years account for 2.4 billion (30%) of the world’s 8.02 billion persons [3].

The sustainable development goals (SDGs) promulgated in 2015 by the United Nations (UN) have laid immense emphasis on youth development for a sustainable future. The goals related to youth are: Goal 4 (Quality Education) and Goal 8 (Decent work and environment) [4]. Youth2030 is the UN Youth Strategy to enhance youth participation and its third progress report published in 2023 suggested that there has been some improvement in youth engagement, however, persistent challenges remain regarding meaningful participation, understanding diversity across youth, and listening to the most marginalized young people. At the international level, the UN plans to address these challenges through the establishment of a UN Youth Office, among other institutional changes. However, it is also important to promote meaningful engagement at regional, national, and local levels. India has one of the highest rates of young people not currently in education, employment, or training (27.2%), high youth unemployment (8.2%) and worrying trends related to youth depression, alcohol consumption and suicide [5].

India being the most populous country in the world is home to about 20% of the world's population, out of which 65% is below the age of 35. Being the youngest country in the world, 20% of the Indian population is below the age of 10 years and the adolescent population stands at 20% of the total [6]. The challenges confronting the huge numbers of adolescents have captured the attention of policymakers as well. This revelation has led to the inclusion of adolescents in the Reproductive Maternal Neonatal Child Health (RMNCH) program of the Government of India as the plus A (RMNCH+A) and also the rolling-out of a youth-specific program, i.e. Rashtriya Kishor Swasthya Karyakram (RKSK) [7]. Research has shown that the influence of a spiritual teacher (or Guru) leads to higher levels of spirituality in seekers [8]. Studies have also shown that through moral instruction, character would be developed and additional benefits, such as better behaviour in the classroom, higher academic performance and increased societal justice, would be achieved [9].

Amartya Sen, Nobel laureate argued for five components in assessing capability [10], one of them being a balance between materialistic and non-materialistic factors in measuring human welfare. The non-materialistic factors can be summed as the spiritual health of the person concerned. Previous authors operationalize spirituality as viewing life in new and better ways, adopting some conception as transcendent or of great value, and defining oneself and one’s relation to others in a manner that goes beyond provincialism or materialism to express authentic concerns about others [11,12]. There is paucity of literature as far as youth spirituality is concerned, with few studies on undergraduate students from South India [13,14]. The relative paucity could be due to increased ethical demands of gaining parental permission for research and that of school systems and staff, as well as that from young people themselves [15]. Developing instruments with language that is appropriate for young people has also proved a challenge [15]. The present study was conducted to generate baseline information on spiritual health and positive youth development of adolescents living in urban areas of Northern India and also to validate the spiritual health questionnaire for use among Indian adolescents.

## Materials and methods

A cross-sectional study was done from June 2019 - May 2020 in an urban area of Northern India. The inclusion criteria were all middle and late adolescents aged 15-19 years residing in the study area for at least six months and giving valid assent/consent for the study. The exclusion criteria were not giving assent/consent for the survey or being non-cooperative. From the entire accessible population (around 30,000), 300 adolescents were selected using non-probability purposive sampling. The sample size of 300 was based on a 36% prevalence of good spiritual health among adolescents, relative error of 20%, design effect of 1.6, and non-response rate of 10% [13].

A standard procedure for recruitment and assessment was used for data collection. For the recruitment of participants, the entire urban field practice area of a tertiary teaching hospital was taken. There were 30 Anganwadi centres (AWC) in the area, each catering to a population of 1,000. Anganwadi centres are established by the government to provide predominantly maternal and child health services, like immunization, supplementary feeding and non-formal education. In addition to these services, the adolescent residing in the catchment area are also provided health interventions. 

The adolescents visiting the centres as well as those residing in the catchment area of the centre were approached for the survey. Participants were recruited from AWCs and local youth groups situated in the same area. Following initial contact, a meeting with the selected adolescents/youth groups was arranged in which the aims and objectives of the survey were conveyed. Participant Information Sheets (PIS) and Participant Informed Consent Form (PICF) were distributed to adolescents aged 18 and above, and informed consent was taken for the survey. After written consent was provided in the PICF, the questionnaires were distributed in the local language. 

Participants who were below 18 years old were asked to take the information sheets and consent forms home to their parent(s)/legal guardian(s) to obtain parental consent. Participants were asked to return the signed consent form to their AWW/youth group leader. Next day when the adolescent returned with parental consent, verbal assent was taken by reading out the assent form to them in the local language and then questionnaires were distributed. Only after the parents gave consent, child's assent was taken.

The questionnaire consisted of socio-demographic questions, the measure of spiritual health (The Index of Core Spiritual Experiences or INSPIRIT tool) and measure of Positive Youth Development Short-Form (PYD-SF). The INSPIRIT and PYD-SF questionnaire took approximately 20 minutes to complete. If the adolescents needed any assistance for completing the questionnaire on their own, the investigators provided assistance using read-aloud techniques and clarifying the queries. After taking part, all participants were debriefed and encouraged to ask questions about the research.

The INSPIRIT Questionnaire had 18 questions, six related to spiritual beliefs and 12 related to experiences, and each question is rated on a scale from 1 to 4. The scores on the first six questions and the highest score on the last 12 questions were summed to obtain the total score ranging from 7-28 [16]. The PYD-SF tool consisting of 34 questions was administered to the same participants to measure determinants of spiritual health, especially the five Cs: competence, confidence, character, connection, and caring [17,18]. It consisted of six questions each measuring competence, caring, and confidence and eight questions each for measuring character and connection. 

Although great care was taken to include adolescents who differed in age, gender, and socio-economic status, the study was conducted with a purposive sample. The primary outcome measures for adolescents included the spiritual health score (INSPIRIT score). The data was entered and analysed using SPSS (Statistical Package for Social Sciences) version 20.0 (IBM Corp., Armonk, NY, USA). Descriptive statistics like frequencies and percentages were used to describe the characteristics of the study population. Mean and standard deviations were used for continuous variables. Analysis of variance (ANOVA) test was done to see the association between study population characteristics and outcome measures. Odd’s ratio (OR) with 95% CI was calculated. Continuous variables were compared using independent t-test and correlations were performed using Pearson product-moment correlation. Exploratory factor analysis (EFA) was conducted to determine whether individual items clustered on the seven-item or 18-item construct. Since many of the underlying constructs are somewhat similar, a Varimax rotation with Kaiser Normalization was applied allowing items to correlate with each other [19]. Binary logistic regression was used to determine predictors of spiritual health, by using age, gender, parental education, and PYD constructs as independent variables. The assumptions of linearity, independence, no multi-collinearity, and equal variances were tested.

## Results

Out of 300 study participants, 157 (52.3%) were males and 143 (47.6%) were females (Table 1). The age of the participants ranged from 15-19 years (middle and late adolescents) with a mean of 17.8 (±0.9) years. The median and mode of age in both males and females was 18 years respectively. The majority i.e. 283 adolescents (94.3%) were professing Hinduism in comparison to only 17 (5.6%) who practiced other religions (Table 1).

**Table 1:** Socio-demographic characteristics of the study participants (N=300)

Age (in years)	Male (%)	Female (%)	Total (%)
15	1 (0.6)	0	1 (0.3)
16	17 (10.8)	10 (6.9)	27 (9.0)
17	39 (24.8)	34 (23.8)	73 (24.3)
18	57 (36.3)	70 (48.9)	127 (42.2)
19	43 (27.4)	29 (20.3)	72 (24.0)
Religion			
Hindu	151 (96.2)	132 (92.3)	283 (94.3)
Others	6 (3.8)	11 (7.7)	17 (5.6)
Language			
Hindi	135 (85.9)	115 (80.4)	250 (83.3)
English	11 (7.0)	19 (13.3)	30 (10.0)
Others	11 (7.0)	9 (6.3)	20 (6.7)
Parents’ Highest Educational Qualification			
Primary	21 (13.4)	0	21 (7.0)
Secondary	20 (12.7)	4 (2.8)	24 (8.0)
Pre-University	20 (12.7)	17 (11.9)	37 (12.3)
Graduate	51 (32.5)	58 (40.6)	109 (36.3)
Post Graduate	45 (28.7)	64 (44.8)	109 (36.3)
Current living status			
With biological parents	86 (54.7)	60 (41.9)	146 (48.7)
Residential facility (Rent/Hostel etc.)	61 (38.9)	80 (55.9)	141 (47.0)
With biological mother/father	5 (3.2)	2 (1.4)	7 (2.3)
With adoptive parents	5 (3.2)	1 (0.7)	6 (2.0)
Total	157 (52.3)	143 (47.6)	300

Most participants i.e. 250 (83.3%) were Hindi speaking followed by English (10.0%). Only 7% of participants reported their parents as primary educated, while three-fourths (72.6%) of the respondents had parents who were graduate or post-graduate (Table 1). The normality testing of INSPIRIT scale using Shapiro-Wilk test revealed that kurtosis values of the items range from -1.48 to 2.41, therefore no item was substantially kurtotic. In addition, all items showed skewness within an acceptable range (univariate skewness less than 3.0). Literature suggests that skewness values less than 2 and kurtosis values less than 7 can be accepted to be within the cut-off for normal distribution [20].

**Figure 1:**
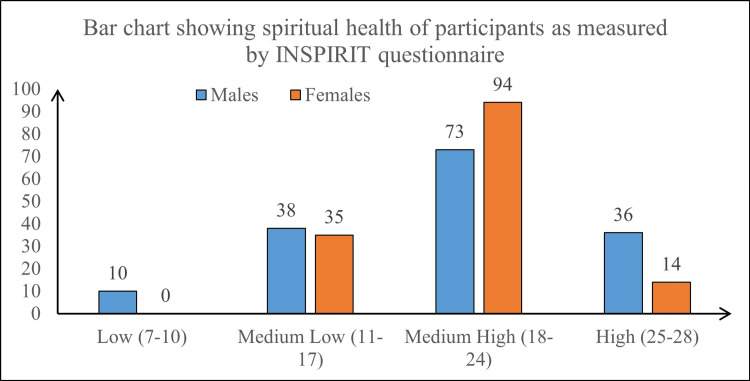
Bar chart showing the spiritual health of study participants based on gender (N=300) INSPIRIT: Index of Core Spiritual Experiences

As far as spiritual health is concerned, 167 (55.7%) of the study participants scored medium-high, followed by 73 (24.3%) of the participants who scored medium-low on the spiritual health scale using the INSPIRIT Questionnaire (Figure 1). The mean score was 19.87 (±4.49) with the full range of scores from a minimum of 7 to a maximum of 28.

The highest mean score on spiritual health scale was obtained in the item concerning spiritual experiences (3.28 ± 0.80) followed by God dwells within (3.14 ± 0.84) and God’s existence item (3.08 ± 1.00). The INSPIRIT tool had sound psychometric properties, demonstrated by good internal reliability statistics. The Cronbach’s alpha for the scale was 0.832 (0.797-0.863) indicating good reliability of the measure.

Univariate analysis showed that spiritual health increased from female to male gender and varied with parental education; however, the difference based on gender was not statistically significant (p value = 0.87). The mean spiritual health score in males was 19.92 (± 5.27), whereas in females it was 19.83 (± 3.61). The mean spiritual health score in adolescents whose parents were primary educated was 22.19 (± 3.53), whereas the score in adolescents with post-graduate parents was 18.66 (± 4.82). On applying analysis of variance (ANOVA) test, the difference was also found to be statistically significant (F stat: 3.43, p value=0.005), indicating an association between parental education and spiritual health of adolescents.

The scores on the INSPIRIT tool were checked for normality using Shapiro-Wilk and Kolmogorov Smirnov tests and were found to violate the assumption of normality. Hence, the interval scale data was transformed into binary variable using a cut-off of 18, so that lower and higher scorers can be differentiated. The multivariate analysis using binary logistic regression showed that positive traits like caring (OR 1.19, 95% CI: 1.08-1.33), connection to school (OR 1.16, 95% CI: 1.04-1.29), having positive identity (OR 1.19, 95% CI: 1.04-1.36) and having highly educated (post-graduate) parents (OR 2.18, 95% CI: 1.13-4.21) lead to significantly higher probability of having spiritual health scores more than 18 (Table 2).

**Table 2:** Multivariate analysis showing determinants of spiritual health among study participants *CI: Confidence Intervals, PYD: Positive Youth Development

Groups	Predictors	Beta	Odds Ratio (95% CI)	Wald (χ2)	p value
Gender	Male	-.38	.69 (.37-1.27)	1.44	.230
	Age	.19	1.21 (.92-1.59)	1.92	.165
Parental Education	Primary				
	Secondary	1.52	4.56 (.84-24.64)	3.11	.078
	Pre-university	1.47	4.37 (1.09-17.53)	4.32	.038
	Graduate	.90	2.47 (.93-6.56)	3.28	.070
	Post Graduate	.78	2.18 (1.13-4.21)	5.33	.021
PYD Constructs	Caring	.18	1.19 (1.08-1.33)	11.14	.001
	Connection to school	.15	1.16 (1.04-1.29)	7.55	.006
	Positive identity	.17	1.19 (1.04-1.36)	6.58	.010

**Figure 2:**
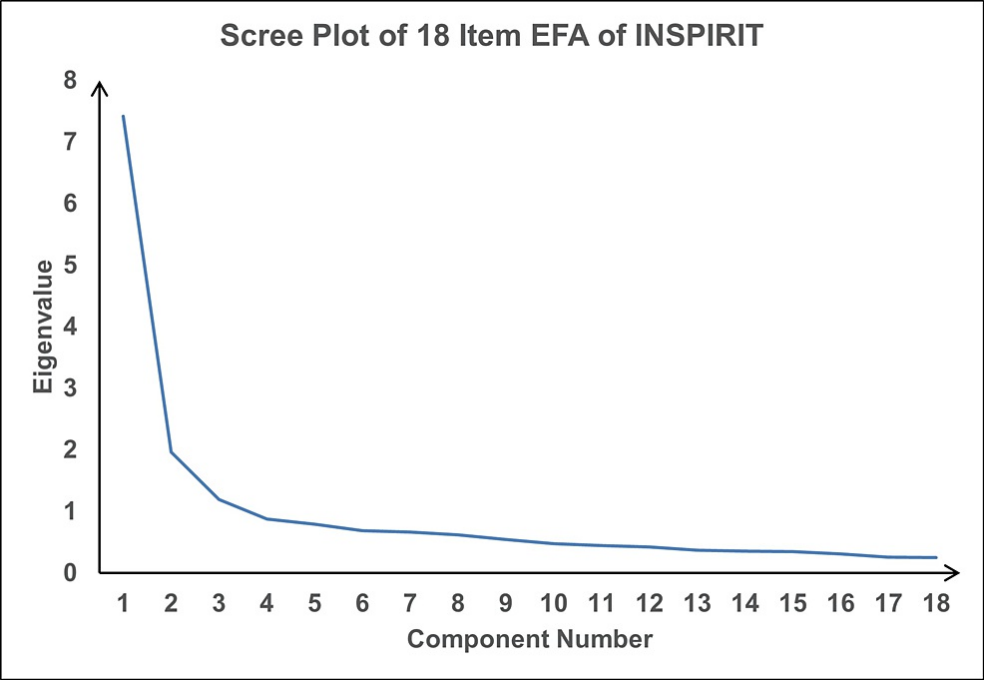
Scree plot of eigenvalues after performing exploratory factor analysis (EFA) INSPIRIT: Index of Core Spiritual Experiences

When including seven items in the EFA, a principal component analysis (PCA) followed by varimax rotation created a one-factor solution with an eigenvalue of greater than one (3.55), explaining 50.7% of the variance. When all 18 items were included, EFA produced three factors with eigenvalues above one, but only three items loaded on the last factor with loading greater than 0.5 value. When the number of factors was limited to three, eight items related to spiritual experiences loaded on the first factor, six items (Item 1-6) on the seven-item INSPIRIT loaded on the second factor and the last three spiritual experiences loaded on the third factor. This resulted in three components with eigenvalues of 1.0 or higher that accounted for 58.7% of the total variance explained (Table 3, Figure 2).

**Table 3:** Exploratory factor analysis (EFA) of the seven Items in the INSPIRIT questionnaire. * Extraction Method: Principal Component Analysis. Rotation converged in six iterations. INSPIRIT: Index of Core Spiritual Experiences

Questions in INSPIRIT Scale	Seven-item EFA	18-item EFA		
	Factor Loading	Factor 1	Factor 2	Factor 3
1. How strongly religious are you?	0.606		0.730	
2. How often do you spend time on religion?	0.682		0.817	
3. How often have you felt close to God?	0.553		0.652	
4. How close do you feel to God?	0.610		0.631	
5. Have you had any spiritual experience?	0.548		0.586	
6."God dwells within you".	0.433		0.509	
7. Spiritual Experiences	0.540			
A. An experience of profound inner peace		0.637		
B. An overwhelming experience of love		0.435		
C. A feeling of unity with the earth and all living beings		0.677		
D. An experience of complete joy and ecstasy		0.744		
E. Meeting or listening to a spiritual teacher or master		0.678		
F. An experience of God's energy or presence		0.730		
G. An experience of a great spiritual figure		0.705		
H. A healing of your body or mind (or witnessed it)		0.708		
I. A miraculous (or not normally occurring) event		0.631		
J. An experience of angels or guiding spirits				0.686
K. An experience of communication with someone dead				0.862
L. An experience with near death or life after death				0.832

## Discussion

The goal of the current study was to find the psychometric properties of the INSPIRIT tool in the Indian population and to determine the level of spiritual health, especially among urban adolescents. Furthermore, the anthropomorphic approach in the study aimed to extend the measurement of spiritual health by finding its determinants in an Indian context. These goals are important to provide a practical measurement instrument of spiritual health in India. Most of the literature on adolescent spirituality concerns its protective influence on risk-taking behaviour as well as end-of-life care with terminal illnesses, like cancer etc. [21,22].

In the current study, 157 participants (52.3%) were males and 143 (47.6%) were females. Age of the participants ranged from 15-19 years (middle and late adolescents), with the majority professing Hinduism. This is in conformity with the religious distribution of Delhi state where Hindus represent 84% of the population followed by Islam and Sikhism [23].

The male percentage was slightly higher than females indicating the adverse sex ratio of urban India. Although the sampling technique was not random, the study enrolled fewer females than males. Out of 300 participants in the current study, 199 (66.3%) were late adolescents and 101 (33.7%) were middle adolescents. The greater representation from later age groups was purposeful because a Likert-type study tool requires proficiency and dexterity on the part of the adolescents. In the current study, the tool had a Cronbach’s alpha of 0.832. Previous studies have reported Cronbach’s alpha ranging from 0.79-0.85 [24].

Using adjusted analysis, no association was found between spiritual health and variables like age, body mass index (BMI) or gender. Previous studies had demonstrated that self-rated importance of spiritual health, both overall and within most questions and domains, declined as young people aged [25]. The current study found a decline in spiritual health with age, but the difference was not statistically significant. However, a statistically significant association was found between parental education and spiritual health indicating a positive association between the two. This finding corroborates numerous previous studies that reported positive association between parental education and a child’s mental and spiritual health, primarily mediated by the parents' spiritual intelligence level [13,26]. This finding highlights the fact that parental education broadens the adolescents’ perspective and encourages spiritual growth and faith in a higher power. 

In the current study, most of the participants scored >18 (medium-high to high) on the INSPIRIT questionnaire. The positive predictors of scoring >18 were found to be positive personality traits like caring for others, having a strong sense of connection to school and being confident of having a positive identity. The personality traits related to the five Cs (character, competence, caring, connection, and confidence) were measured using the PYD-SF tool, which has been used to measure positive youth traits among adolescents. Previous studies have reported that most adults from test sites score between 16 and 25, with a mean of 20.5 [23]. Previous studies have also hypothesized that religiosity and spirituality may affect adolescent health and well-being through a variety of processes that influence attitudes, values, skills, constructive use of time, and social relationships [27]. Keeping track of long-term goals, despite opportunities for short-term rewards, is regarded as an important predictor of positive development in adolescents [28]. 

In this study, the INSPIRIT items were analyzed as seven items or 18 items to determine whether or not they fit the underlying constructs. As described above, a second scoring method was used which included all responses to the 12-part checklist. The factor analysis using the seven items produced only one factor, whereas the 18-item checklist produced three factors with eigenvalues above one, but only three items loaded on the last factor. When the number of factors was limited to three, these three items again appeared as the only items in the third factor. Once it was determined which items contributed to the latent construct, the items were organized into subscales using EFA. This is a technique designed to explore the underlying structure, based on the patterns in the theory. These techniques led to the conclusion that important information was lost by merging the last 12 items into one, as per the recommendations of Kass et al. [16].

The current study had few limitations. First, a non-probability purposive sampling limits the generalizability of the findings, and self-reported design leads to social desirability and recall biases. Second, the sample was limited to middle and late adolescents, who might rate the tool differently from early adolescents and children. Hence, a comprehensive assessment of all age groups is further warranted for understanding the effect of age on spiritual health and PYD scores. The most important challenge is the difficulty in gauging the experiences and cognitive elements of a 'spiritual' event which is an internalized relationship between the individual and 'God'; not all adolescents may have had this experience or developed this close relationship with God. However, the study still adds significant scientific evidence as it tried to determine the predictors of spiritual health using multi-variable adjusted analysis, using PYD constructs as independent variables.

## Conclusions

The spiritual health of most adolescents was medium-high to high, and the major determinants were related to positive youth development traits. Also, multi-variate analysis revealed a positive association with positive youth development traits like caring and confidence. This study provides a useful measurement instrument of youth spirituality in India. The INSPIRIT tool construct was found to have good psychometric properties in an Indian setup and can be used as a theoretical framework for promoting adolescent development. This was a small-scale study, and further scaling-up is needed to generate more evidence on spiritual health, among various age groups. The study was unique as it employed an anthropomorphic approach to study spiritual health, by finding its determinants with respect to positive youth traits. There is a need to generate greater political support and commitment for spiritual education. Further studies are required to determine how effective spiritual education programs for behaviour change can be designed to be sensitive and meaningful across diverse cultures and religions.
